# Electronic learning can facilitate student performance in undergraduate surgical education: a prospective observational study

**DOI:** 10.1186/1472-6920-5-23

**Published:** 2005-06-29

**Authors:** David Gerard Healy, Fergal J Fleming, David Gilhooley, Patrick Felle, Alfred Edward Wood, Thomas Gorey, Enda W McDermott, John M Fitzpatrick, Niall J O'Higgins, Arnold DK Hill

**Affiliations:** 1 Department of Surgery, Mater Misericordiae University Hospital, Eccles St, Dublin 7, Ireland; 2 Department of Surgery, St Vincent's University Hospital, Elm Park, Dublin 4, Ireland; 3 Conway Institute of Biomolecular & Biomedical Research, University College Dublin, Dublin 4, Ireland; 4 Centre for Health Informatics, University College Dublin, Dublin 4, Ireland

## Abstract

**Background:**

Our institution recently introduced a novel internet accessible computer aided learning (iCAL) programme to complement existing surgical undergraduate teaching methods. On graduation of the first full cycle of undergraduate students to whom this resource was available we assessed the utility of this new teaching facility.

**Method:**

The computer programme prospectively records usage of the system on an individual user basis. We evaluated the utilisation of the web-based programme and its impact on class ranking changes from an entry-test evaluation to an exit examination in surgery.

**Results:**

74.4% of students were able to access iCAL from off-campus internet access. The majority of iCAL usage (64.6%) took place during working hours (08:00–18:00) with little usage on the weekend (21.1%). Working hours usage was positively associated with improvement in class rank (P = 0.025, n = 148) but out-of hours usage was not (P = 0.306). Usage during weekdays was associated with improved rank (P = 0.04), whereas weekend usage was not (P = 0.504). There were no significant differences in usage between genders (P = 0.3). Usage of the iCAL system was positively correlated with improvement in class rank from the entry to the exit examination (P = 0.046). Students with lower ranks on entry examination, were found to use the computer system more frequently (P = 0.01).

**Conclusion:**

Electronic learning complements traditional teaching methods in undergraduate surgical teaching. Its is more frequently used by students achieving lower class ranking with traditional teaching methods, and this usage is associated with improvements in class ranking.

## Background

Medicine has become increasingly complex and the challenges faced by the medical education system are becoming even greater. Higher levels of technical and scientific knowledge are required as well as effective communication and management skills. Acquisition of this knowledge must be achieved within a finite time period. In addition the clinical opportunities for medical students are diminishing with decreasing length of hospital stay for many surgical procedures. This environment has led to the search for novel teaching methods to deliver undergraduate medical education.

Computer aided learning (CAL) offers distinct advantages over conventional teaching methods, including the potential for multimedia applications with a significant interactive content. Not only may text be presented, but in addition, tables, images, video and animation can be integrated into dynamic packages. In this manner multimedia education strategies offer potential strategic advantages over traditional paper based material. The system is very flexible allowing a lecturer to update and upload data in the package and to monitor an individual student's activity. An internet-based computer aided learning (iCAL) programme has the additional advantage of access from an off-site location at any hour of the day. Unfortunately the establishment of the infrastructure for iCAL is quite costly and the cost to benefit analysis of undergraduate iCAL has been questioned[1] The Department of Surgery at University College Dublin, Ireland, has recently introduced an iCAL package for undergraduate education. We wished to evaluate the effectiveness of this programme following the first complete cycle of undergraduate medical students.

## Methods

Medical undergraduates at University College Dublin (UCD) have a forty two week training period in surgery. This is divided in two parts, a 28-week period and a 14-week period. Students are allocated clinical attachments to hospital surgical services and rotated though the spectrum of surgical specialities. During these rotations they participate in ward, theatre and outpatient activities but have no clinical responsibilities. Clinical "by the bedside" teaching with clinical lectures in small groups is provided. In addition a formal lecture programme is delivered and library facilities are provided for independent learning. This structure is the same in the initial 28 week and the later 14 week periods. The clinical sites are identical, and clinical rotations are organised so that all students get an exposure to all surgical specialties over the course of their training over the entire 42 week period. Whether an individual student goes through a particular rotation in the initial 28 weeks or the later 14 weeks, is a random allocation.

At the end of the first period of the programme, undergraduates have a surgical examination consisting of a multiple choice paper and a clinical examination. For the purposes of this paper this is referred to as the entry examination. At the end of the second period of the course students take the final exit examination in surgery. The exit exam also consists of a multiple choice exam and clinical exam with an additional written short answer examination.

iCAL-SURG was used during the second period of the surgery course, after students had taken their entry examination. The only fundamental methodology difference between these two training periods was the introduction of the iCAL programme. The questions presented through the iCAL system are identical in style to those presented in the short written examination and clinical examination problem solving scenarios.

The class was divided into quartiles on the basis of this entry examination. Utilisation of the iCAL programme by students was optional and was compared with absolute performance and change in class ranking in the final examination. One hundred and forty eight students completed the two year programme. Data on these students' utilisation of the iCAL platform was analysed.

### Technical infrastructure

All students in UCD Medical Faculty are conversant with use of ICT, with CAL used to deliver up to 35% of preclinical courses. Clinical students have access to a suite of networked high specification multimedia PC's with T1 internet access. This facility is available to students from 8.00 to 18.00, weekdays. iCAL-SURG is also available anywhere with an internet connection. Online course material is delivered within the Blackboard  Virtual Learning Environment. Courses are password protected and use of iCAL-SURG by individual students is monitored. Instructors can find the number of times each student has logged on to iCAL-SURG, the areas of the course accessed, the time and day of log-on. The number of times the site was accessed by the student is referred to as the "hit rate". It is not possible to record the content viewed or duration of student activity, once logged on.

### iCAL-SURG

During this pilot phase, iCAL-SURG use was optional and did not form part of the assessment process for students. This phase concentrated on the COURSE DOCUMENTS and the ASSIGNMENTS areas in Blackboard. The COMMUNICATION area was used for both social and academic purposes but only accounted for 5.3% of use of iCAL-SURG and no assessment was made of this in relation to student performance.

In the COURSE DOCUMENTS area [Fig, 1,2] lecture notes are available prior to lectures, either as Powerpoint, Adobe Portable Document Format (PDF) or Microsoft Word files. The material available on the iCAL system not only included material presented at lectures but also additional supplementary material. Old examination question papers and sample answers are also available here. In the ASSIGNMENTS area each week the academic staff uploaded a short series of clinical questions with suggested answers made available made available [Fig. 3]. Students read these and studied the relevant areas in text books or in discussion with tutors and peers. Students could also take online OSCE examinations [Fig. 4,5]. This was presented as a series of photographs with related clinical details. Students were asked to answer the questions in a prepared downloaded Microsoft Word document, which they then uploaded to the "Digital Dropbox" in Blackboard. These submissions were reviewed by academic staff. On the following day sample answers were published [Fig. 4,5] which students could compare with their submissions.

**Figure 1:**
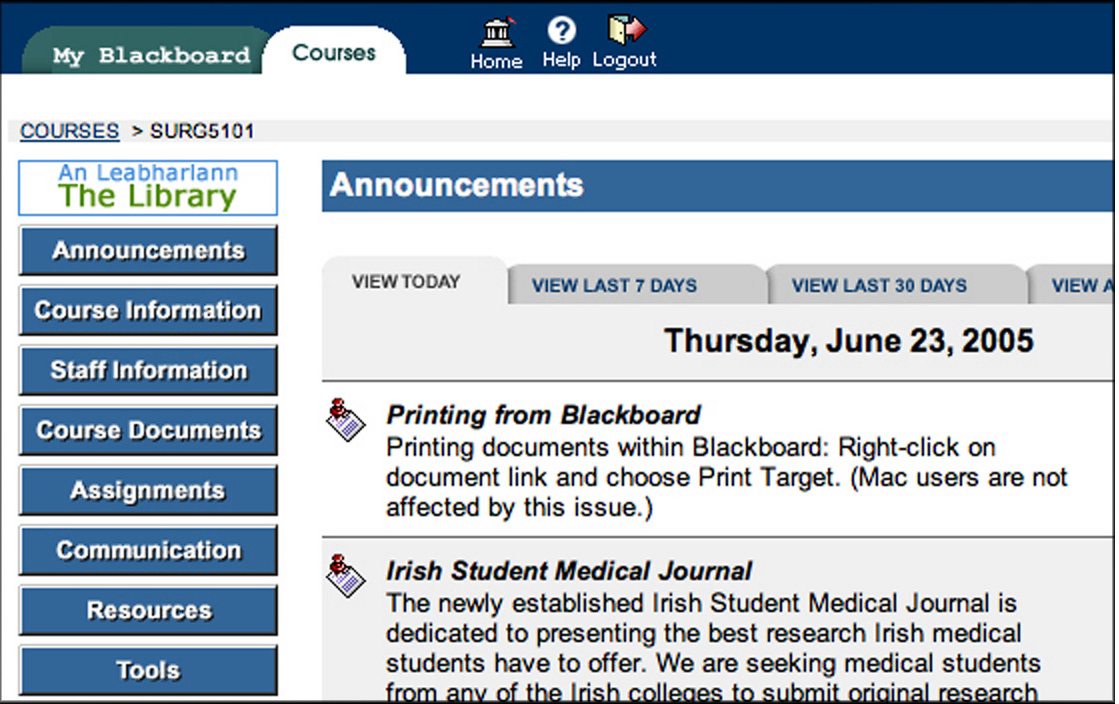
Screenshot from iCAL-SURG.

**Figure 2:**
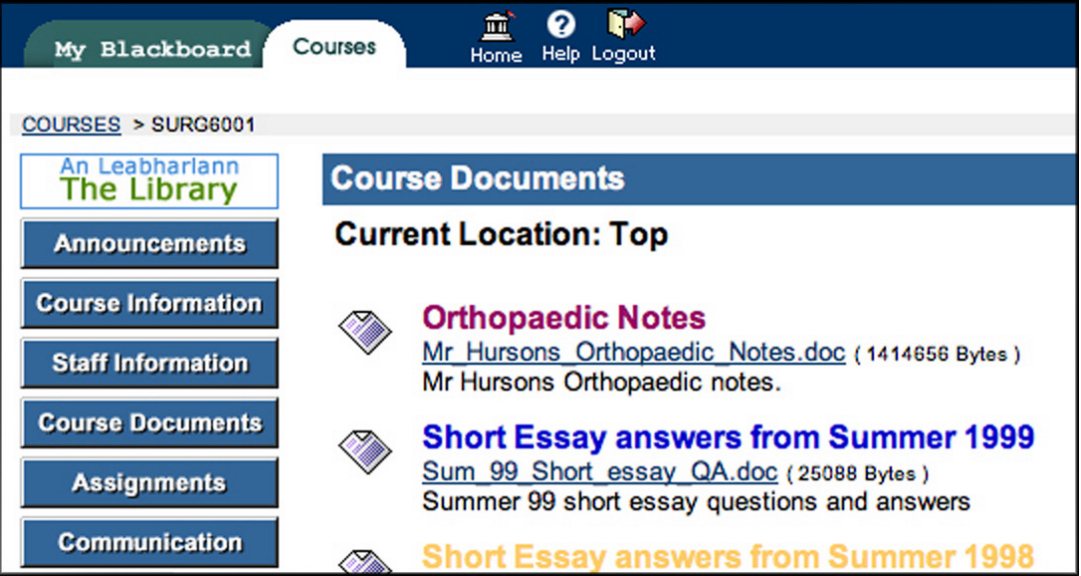
Screenshot taken from some materials available in course materials.

**Figure 3:**
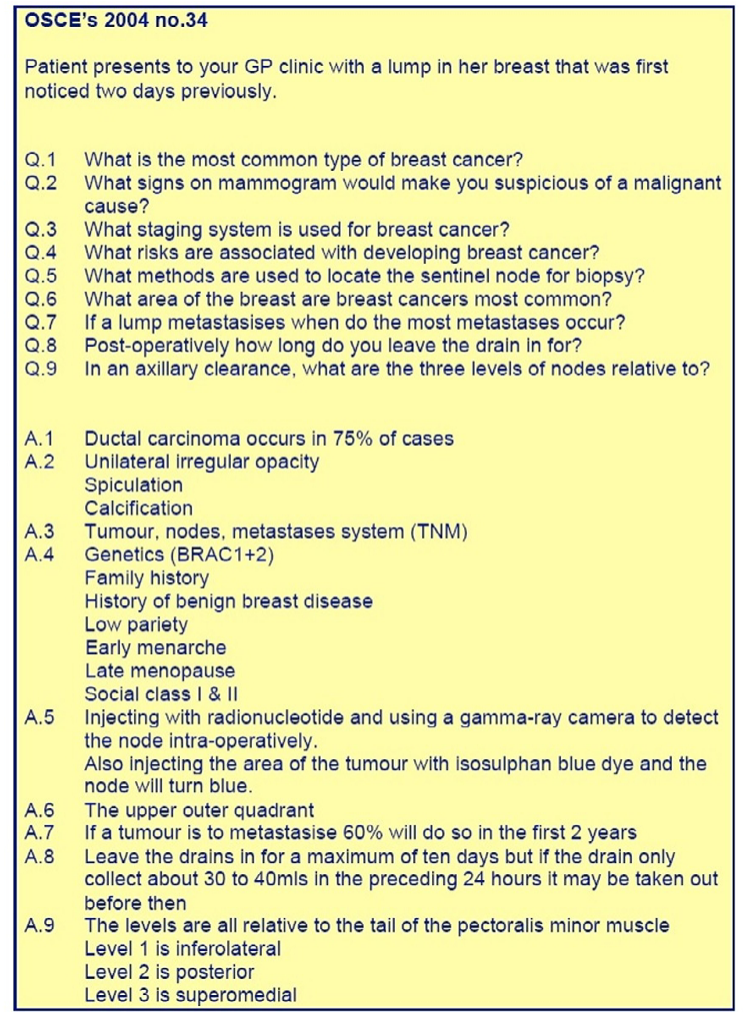
This is an example of a short OSCE available on iCAL-SURG.

**Figure 4:**
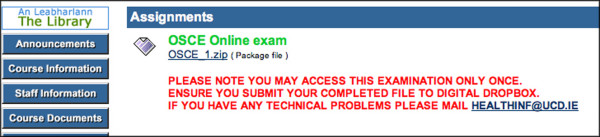
Screenshot of link to OSCE with instructions.

**Figure 5:**
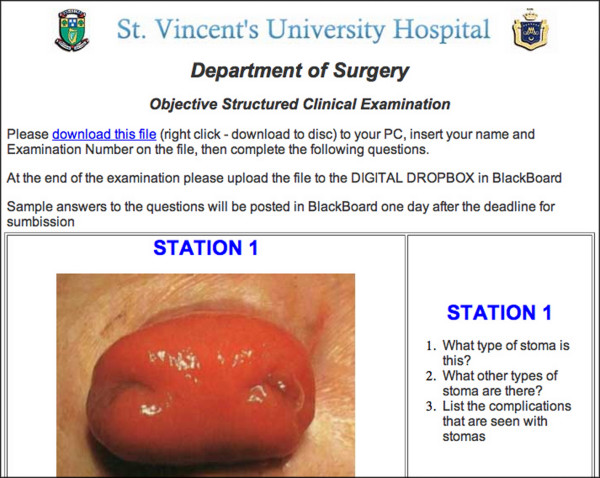
Screenshot of first station in a full online OSCE.

### Statistical analysis

A Student t test was used for comparison between groups of two. Where more than two groups were involved an ANOVA was performed followed by Post-hoc analysis as appropriate, with a Fischer's least significant difference test. Data involving class rank was analysed non-parametrically with a Mann Whitney U Test for comparison between groups of two, a Kruskal Wallis where more groups were involved and a Spearman Rank Correlation for investigating correlations. Alpha was set at 0.05.

## Results

**Figure 6:**
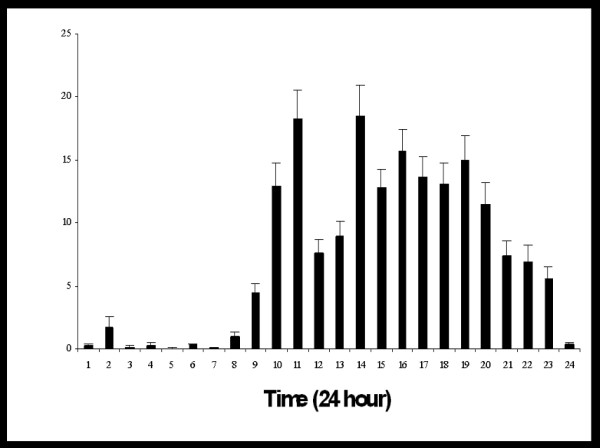
This shows the pattern of computer usage over the day. Data is presented as means with standard errors.

**Figure 7:**
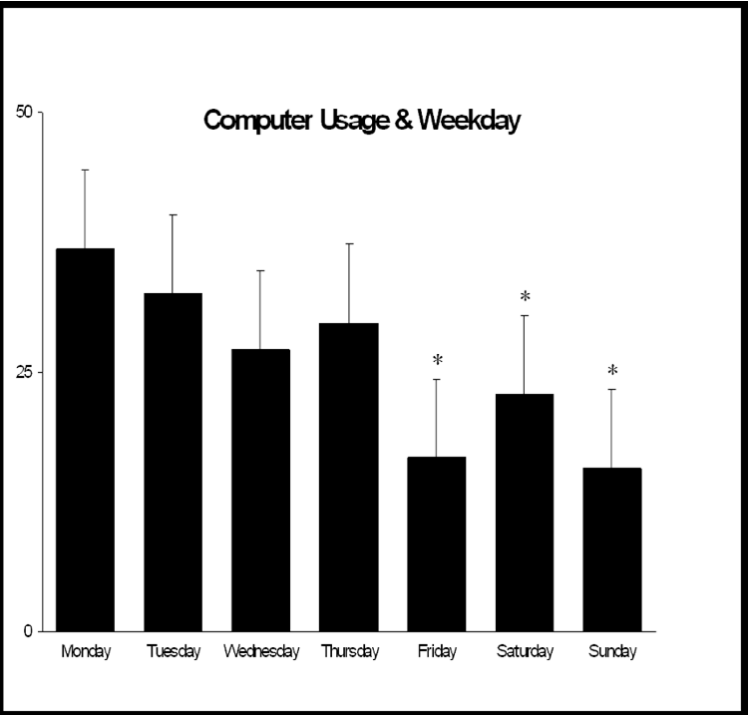
This shows the number of hits into the computer system each day. Use of the system on a Friday, Saturday or Sunday was significantly less than use on Monday, Tuesday and Thursday (ANOVA P < 0.001). * P < 0.05

Complete data on computer utilisation was available on 148 students. There were 58 males and 90 females. There were no significant gender differences in the utilisation of online access (P = 0.3) or final year examination results (P = 0.251). One of the criticisms of the programme from the start was the concern that students without independent computer and internet access would be disadvantaged. 74.4% of students were able to access iCAL from off-campus internet access. Despite this high availability of independent internet access, the majority of iCAL usage (64.6%) took place during daytime hours (08:00–18:00) [Fig. 6] with less then expected usage on the weekend (21.1%) [Fig. 7].

To assess the educational benefits of the programme the class was ranked based on the entry test results. Class rank on the entry test was then compared to the class rank at the exit exam. Usage of the iCAL system during daytime hours was positively associated with improvement in class rank (P = 0.025) but out-of hours usage was not (P = 0.306). Usage during weekdays was associated with improved rank (P = 0.04), whereas weekend usage was not (P = 0.504). There was no significant difference in improvement in class rank between students with independent internet access and those dependant on access provided by the University Hospitals. We therefore felt that the computer facilities provided by the University were adequate for the needs of the students and that students without independent computer and internet access were not disadvantaged.

**Figure 8:**
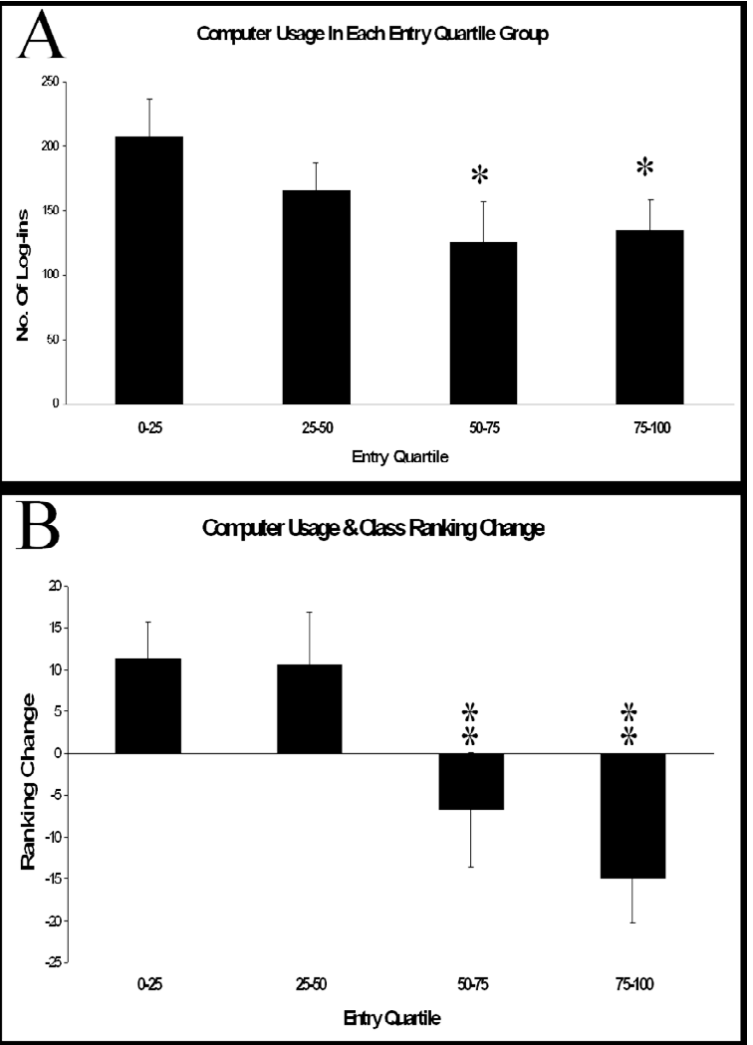
Students have been divided into quartiles based on the performance in the entry surgical examination. A] This shows the usage of the surgical computer education package by students in different class rank quartiles. Significantly more use was made of the computer package by students in the lowest entry rank quartile (ANOVA P = 0.042). B] This shows the change in class ranking between entry examination and final examination in surgery. Initially low ranking students showed the greatest improvement in their class position (Kruskal Wallis P = 0.01). Mean and standard error. * P < 0.05, ** P < 0.01; compared to lowest class quartile at entry test.

We were also interested in the level of usage of iCAL by different student abilities. Students with lower ranking on the entry test were found to subsequently use the computer system more frequently (P = 0.01). The lower quartiles of the class were the most frequent users of the programme (ANOVA, P = 0.042) [Fig. 8]. We then wanted to evaluate whether this usage translated into improvements in class rank. Usage of the iCAL system was positively correlated with improvement in class rank from the entry to the exit examination (P = 0.046).

## Discussion

This cohort was the first group to complete their undergraduate surgical training since the introduction of this dynamic iCAL package. Of concern to us in establishing this program was the potential to competitively disadvantage students without independent computer and internet access. This in part motivated the decision not to include iCAL as part of the formal student assessment. However our study shows no such disadvantage. Instead, structured usage of iCAL during normal working time was associated with improved class performance rather then usage out of hours. This suggests that improvement in class rank with iCAL was not facilitated by studying late into the night but rather by a structured and disciplined approach. This conclusion is limited by the fact that the system cannot distinguish a student accessing the system from home during these hours or from the University computer suite. However as the students had other onsite daily activities during the day, with monitored attendance, it seems most likely that this activity occurred from University provided facilities.

One of the objectives of this study was to examine the patterns of usage of the iCAL system. Part of the motivation in using an internet accessible format was the expectation that students could utilise the programme as a distance learning tool. Although 35% of iCAL utilisation took place outside of the normal academic day, this usage was not associated with improvements in relative position in the class. This at first glance questions the benefit of an internet based infrastructure. However this technology has other advantages to a faculty using more then one teaching hospital, such as enabling the educators to upload data from multiple sites.

We were also interested in the utilisation of the programme by students of different class rank. It was interesting to us that the top two quartiles of the class, as ranked by the entry examination, did not utilise the iCAL programme as frequently as the lower two quartiles. The factors underlying this are uncertain. The method of assessing usage was the number of times an individual student logs into the system. The package does not measure the amount of time spent with the package or the volume of content covered by the student. It is conceivable that students at the lower end of the class took longer to completely digest the content of the web pages and required more frequent visits to the same sites. However if that is the case it appears to have been rewarding as the lowest 25% of the class on the entry test, who also used the iCAL package most, were the group who made the most progress up the class ranking. It must be observed that those at the top have little room to move up and those at the bottom little distance to fall in terms of class ranking. This could have influenced an outcome measure based on class rank. However the pattern was not only seen with the top and bottom quartile but is also seen in the central 25–75% of the class. Another group has reported finding similar to our experience observing that students performing poorly in the entry examination used the CAL programme most and were the individuals with the greatest improvement in grades, although class ranking was not reported[2] Some report the use of CAL as a remedial tool for those performing poorly in conventional evaluation and show improvement of scores following CAL instruction[3]

Criticisms have been raised that iCAL may not be suitable for students with negative attitudes to computers and a student preference for hardcopy material over computer screen presentation is reported[4,5] Some randomised trials of CAL versus lectures reported superior objective outcome measures in the CAL group although students reported a preference for the traditional lecture structure[6] However a positive attitude to CAL was reported when it was an addition to traditional teaching methods, rather then as a replacement[7] The general response of medical students is positive with the view that CAL is a novel and fun way to learn[8] Medical students have however expressed concern that the delivery of an education programme by computer will compromise the student trainer relationship if the computer supplants other forms of training[8] The direction chosen at our unit was not to replace lectures, but rather to complement them with this additional teaching resource. A negative attitude to computers and CAL may not be an obstruction to successful learning however, as knowledge is increased by usage of a CAL programme even among medical students who reported negative attitudes to computers and CAL[5] Not every report of CAL in medical education is positive and it appears that the style of teaching and stage of clinical development of the student is important to the outcome[9,10] While problem based teaching and clinical simulation are very effective methods of CAL in clinical years, it appears that in the early years of basic science instruction in medicine, a more didactic CAL delivery of material is superior to a problem based presentation[10,11]

Few randomised controlled trials have been performed in CAL medical education and many of those that have been performed look at small subunits of the overall medical corriculum[1] Of the trials that have been performed on CAL, the evidence suggests a high information assimilation rate among medical students[5,10-13] Seabra *et al. *wanted to explore the outcome of replacing lectures completely with a CAL programme and demonstrated that computer aided learning could achieve similar results to standard lectures[14] Some evidence suggests that although the gain in knowledge is similar following computer or traditional instruction, the time required to achieve these similar results is less when the student uses the computer aided instruction[15]

## Conclusion

Our experience shows that electronic learning complements traditional methods in undergraduate surgical teaching. It offers advantages to the teaching staff in the speed and convenience of dissemination of information. For students the establishment of an internet based CAL system offers the advantage of distance learning access outside the working day. However our experience has been that such student utilisation of the programme was not associated with improvements in class rank. The iCAL programme was more frequently used by students with lower class ranking with traditional teaching methods, and iCAL usage by these students is associated with improvements in class ranking. The incorporation of iCAL programmes into undergraduate medical education offers distinct administrative advantages for the teaching staff and more learning opportunities for students with diverse abilities and learning preferences.

## Pre-publication history

The pre-publication history for this paper can be accessed here:


